# The Risk Function of Breast and Ovarian Cancers in the Avrami–Dobrzyński Cellular Phase-Transition Model

**DOI:** 10.3390/ijms25021352

**Published:** 2024-01-22

**Authors:** Anna Zawadzka, Beata Brzozowska, Anna Matyjanka, Michał Mikula, Joanna Reszczyńska, Adrianna Tartas, Krzysztof W. Fornalski

**Affiliations:** 1 Maria Skłodowska-Curie National Research Institute of Oncology (NIO-MSCI), 02-781 Warsaw, Poland; anna.zawadzka.fizyk@nio.gov.pl (A.Z.);; 2 Faculty of Physics, University of Warsaw, 02-093 Warsaw, Poland; beata.brzozowska@fuw.edu.pl (B.B.);; 3 Faculty of Physics, Warsaw University of Technology, 00-662 Warsaw, Poland; 4 Mossakowski Medical Research Institute, Polish Academy of Sciences (IMDiK PAN), 02-106 Warsaw, Poland; jreszczynska@imdik.pan.pl; 5 National Centre for Nuclear Research (NCBJ), 05-400 Otwock-Świerk, Poland

**Keywords:** Avrami equation, phase transition, cancer, carcinogenesis, neoplastic transformation, cancer physics, breast cancer, ovary cancer, fractal

## Abstract

Specifying the role of genetic mutations in cancer development is crucial for effective screening or targeted treatments for people with hereditary cancer predispositions. Our goal here is to find the relationship between a number of cancerogenic mutations and the probability of cancer induction over the lifetime of cancer patients. We believe that the Avrami–Dobrzyński biophysical model can be used to describe this mechanism. Therefore, clinical data from breast and ovarian cancer patients were used to validate this model of cancer induction, which is based on a purely physical concept of the phase-transition process with an analogy to the neoplastic transformation. The obtained values of model parameters established using clinical data confirm the hypothesis that the carcinogenic process strongly follows fractal dynamics. We found that the model’s theoretical prediction and population clinical data slightly differed for patients with the age below 30 years old, and that might point to the existence of an ancillary protection mechanism against cancer development. Additionally, we reveal that the existing clinical data predict breast or ovarian cancers onset two years earlier for patients with *BRCA1/2* mutations.

## 1. Introduction

Breast and ovarian cancers (BOCs) are among the most common types of cancer that affect women around the world. According to the latest American Cancer Society report, breast cancer ranks as the second leading cause of cancer-related deaths among women, with an estimated 43,170 patients expected to succumb to the disease in the United States alone in 2024, irrespective of screening or treatment. Ovarian cancer can be more aggressive but the rarer of the two. It ranks fifth in cancer deaths among women.

Although many cases of these cancers are sporadic, there is a subset of individuals who have a genetic predisposition to developing BOCs due to germinal mutations in the *BRCA1/2* DNA repair associated genes. The *BRCA1/2* genes play an important role in DNA repair and the maintenance of genomic stability; mutations in either of these genes can increase the risk of developing BOCs by up to 80% [1]. Most women are diagnosed after the age of 40 or even 50, the time when they start undergoing periodic mammograms. Unfortunately, for hereditary diseases, this age is usually too late for effective treatment. One potential strategy to reduce the global mortality rate is the widespread adoption of more accessible molecular diagnostics—a trend gaining popularity, particularly in more developed countries. Understanding the role of *BRCA1/2* mutations in the development of BOCs is crucial to developing effective screening and prevention strategies, as well as targeted treatments for individuals with these mutations [2,3]. One potential way to tackle this problem is the use of biophysical numerical modeling using age-related BOC incidence data.

Experimental studies of the most common *BRCA1/BRCA2* founder mutations in a population series of BOC cases show that both of those mutated genes differ in the risks of BOC development, the age of onset, and the prognosis of breast cancer [4,5,6]. However, out of several other candidates, these two genes are known to be the most associated with a higher probability of the occurrence of those cancers.

Nevertheless, the full spectrum of breast/ovarian cancer susceptibility genes has not yet been identified, but several of them with strong evidence including *CHEK2* (checkpoint kinase 2 gene), *ATM* (ataxia–telangiectasia mutated gene), *TP53* (tumor protein p53 gene), *PALB2* (partner and localiser of *BRCA2* gene), *PTEN* (phosphatase and tensin homolog gene), and *NBN* (Nijmegen breakage syndrome gene) are considered in screening for hereditary predispositions of those cancers [7]. Dozens of other candidates with relatively medium or weak evidence of breast/ovarian cancer induction such as *XRCC2* (X-ray cross-complementation 2 gene) or *CDH1* (cadherin1 gene) are still under consideration [8]. Above all, *BRCA1* (breast cancer gene 1) and *BRCA2* (breast cancer gene 2) are the most common contributors to breast/ovarian cancers. They are protein-coding genes involved in DNA repair mechanisms, in particular the homologous recombination pathway for double-strand DNA repair. *BRCA1* is located on chromosome 17 (17q21.31) containing 22 exons spanning about 126 kb of DNA. *BRCA2* is placed on chromosome 13 (13q12.3) with a length of more than 85 kb. The genetic analysis of *BRCA1/2* genes identified more than 20,000 of their specific variants (missense/nonsense, frameshift, or large rearrangements). Most of them are rare and strongly dependent on the nationality of a carrier. There are few examples of common variants in Israel (*BRCA1*: c.68_69delAG and *BRCA2*: c.5946delT), Ireland (*BRCA1*: c.427G>T), Iceland (*BRCA2*: c.771_775delTCAAA), Holland (*BRCA1*: c.2804_2805delAA and *BRCA2*: c.5579insA), and Poland (*BRCA1*: c. 68_69delAG) [9].

The management of cancer with precision requires a thorough understanding and accurate analysis of accumulated data. These data include patient information, medical records, imaging results, and molecular profiles. Various analytical methods are used to extract insights from these complex data and guide clinical decision making. The most commonly used statistical methods in the biomedical field allow for the definition of the relationship between variables in datasets. For example, these methods can be used to identify risk factors, assess the effectiveness of treatments, and compare the performance of different diagnostic or prognostic approaches [10]. More recently, machine learning and deep learning algorithms have been increasingly incorporated for clinical imaging and molecular data analyses, and in many instances, they have demonstrated greater accuracy in the diagnosis of various types of cancer than clinicians [11,12].

Over the years, we developed a biophysical model that can be used to describe cancer transformation in the context of the cumulation of oncogenic mutations. Our research aimed to describe clinical data using this theoretical approach, which also allowed for further validation of the model and the proposed interpretation of its parameters. One decade ago, Professor Ludwik Dobrzyński proposed this novel approach in cancer physics: He postulated that the cancer neoplastic transformation of the mutated cell is analogous to the crystal nucleation and growth theory [13,14]. This theory was originally proposed in the 1930s/1940s by Melvin Avrami to describe the physical phase-transition phenomenon for the crystallisation process [15,16,17]. In this context, the biological process of cancer transformation corresponds to the purely physical process of rapid phase transition in condensed matter physics [18]. Although this was not the first proposition to treat carcinogenesis as a form of phase transition [19,20], its novelty is based on the Avrami equation, which can be applied as a probability function of cancer induction [13,14].

The biophysical details of the proposed model and its mathematical derivation were presented in the recent paper by Fornalski and Dobrzyński [14]; therefore, they will not be presented here. However, one needs to know that the calculations start from the well-known biophysical concept of the multi-hit theory of carcinogenesis [21,22]. Next, the Avrami theory of crystal nucleation and growth is taken into consideration and implemented: Each oncogenic mutation is equivalent to the crystallisation cluster. When the number of oncogenic mutations exceeds some critical value (effective threshold), cancer transformation acts as a rapid nonlinear process, analogous to the physical phase-transition phenomenon.

The probability function of that process is given by the so-called Avrami equation (also called Johnson–Mehl–Avrami–Kolmogorov, or JMAK, equation):

(1) $$P\left(m\right)=C\;\left(1-e^{-a\;m^{k}}\right)$$

where *P* is the probability of cancer neoplastic transformation in the population in cells, *m* is the number of oncogenic mutations in DNA, *a* is a shape constant (related to the slope of the sigmoidal curve) responsible for the distribution of mutations, and *k* is related to the cancer transformation dimensionality (*k* = 4 means the 3D development of protein network within cancerous DNA). This equation was applied, and the model was practically tested using real clinical data on gastric cancer, where the results showed *k* = 4.42 ± 0.24, *a* = 0.0085 ± 0.0020, and *C* = 155 [14].

According to very recent findings [23,24], the number of mutations (*m*) is linearly proportional to age (*t*); thus, a premise can be postulated that, with some scaling factor dependent on the percentage of cancer susceptibility genes in the genome, this relationship is also true for the oncogenic mutations considered above.

Assuming the probability normalisation (*C* = 1), one can rewrite Equation (1) to a simpler and more useful form to describe the probability of cancer neoplastic transformation in the population:

(2) $$P\left(t\right)=1-e^{-\alpha \;t^{k}}$$

where *t* is the patient’s age (in years), and *α* is qualitatively equivalent to *a* from Equation (1). This new form allows us to apply the model to a wider number of clinical data, e.g., to simple population statistics of cancer patients, with some limitations related to the pure Avrami’s approach [18].

One must note that according to the original Avrami theory, *k* = 2 means the linear (one-dimensional) development of the crystal, *k* = 3 refers to the two-dimensional one, and *k* = 4 indicates three-dimensional growth [15,16,17]. In the context of carcinogenesis, one can describe the dimensionality of the protein complex network rather than the geometry of the DNA itself. Every *k* > 4 means that this structure is fractal. This scenario will be explained in the Section 3.

## 2. Results

### 2.1. Patients Tested for BRCA1/BRCA2

All clinical data on breast and ovary cancer were collected at the Maria Skłodowska-Curie National Research Institute of Oncology (MSCI) in Warsaw, Poland. All data were divided into 3 groups (datasets), see Table 1.

The first dataset from the MSCI contains 511 clinical cancer cases, in which breast or ovarian cancer was diagnosed (age of diagnosis). Furthermore, all of these patients were tested for the *BRCA1*/*BRCA2* mutation, and 52 cases were positive. Rest datasets do not contain information on mutations.

**Figure 1:**
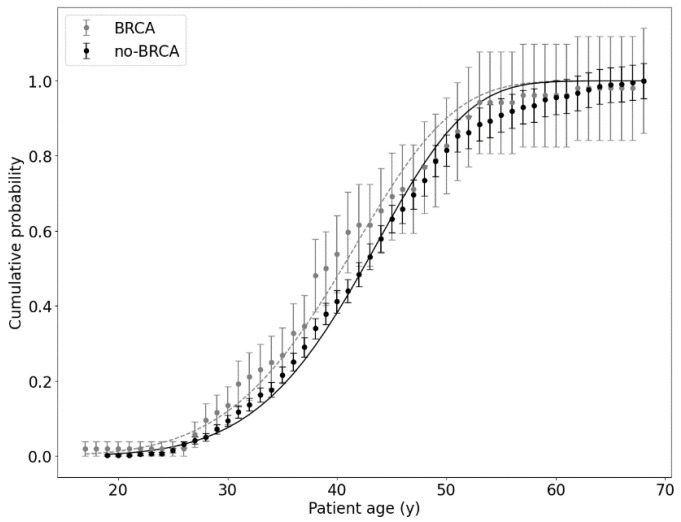
Cumulative probability of cancer diagnosis as a function of patient’s age at the time of diagnosis for the first set of clinical data (511 cancer patients with or without the *BRCA1*/*BRCA2* mutation) calculated with the Avrami–Dobrzyński model, where *α* = (3.6 ± 1.6) × 10^−11^ y^−1^ and *k* = 6.32 ± 0.12 for patients without mutations (black line), and *α* = (5.0 ± 6.0) × 10^−10^ y^−1^ and *k* = 5.69 ± 0.33 for patients with *BRCA1*/*BRCA2* mutations (grey line). All uncertainties represent one standard deviation.

All data from the first dataset, divided into two subgroups (*BRCA* and no-*BRCA* groups), were aggregated to fit the function from Equation (2), with *R*^2^ equal to 0.99 for both datasets. The results are presented in Figure 1.

One has to note that both curves in Figure 1 are regularly shifted. Therefore, an additional parameter *t*_0_ was introduced through the modification of Equation (2) ($P\left(t\right)=1-exp\;\left[-\alpha \;{\left(t-t_{0}\right)}^{k}\right]$). A new function (with fixed *α* and *k* parameters) was fitted to both sets of data, with and without *BRCA1*/*BRCA2* mutations, and parameter *t*_0_ was determined.

The time shift between both curves was calculated as a subtraction of *t*_0_ values for both subpopulations and was equal to (2.29 ± 0.20) years. This shift value, in our understanding, represents the difference in the time of cancer occurrence; therefore, this means that patients with *BRCA1*/*BRCA2* mutations are diagnosed with breast or ovary cancer approximately two years earlier than patients without such mutations. This seems to be quite a natural situation, which can be found in the literature. For instance, in the Latvian study, this period varied from ~9 to even ~16 years [5], while in the Belarussian study, it was equal to 4–5 years [4]. Our results are therefore much closer to those of Belarus and suggest that a new oncogenic mutation can be obtained in breast/ovary somatic cells every two years of human life.

### 2.2. Results for Cancer Patients without Genetic Tests

Two additional datasets from Table 1 cover cancer patients who were not tested against *BRCA1*/*BRCA2* mutations. However, these data are also useful in the context of model validation and the comparison of the results.

**Figure 2:**
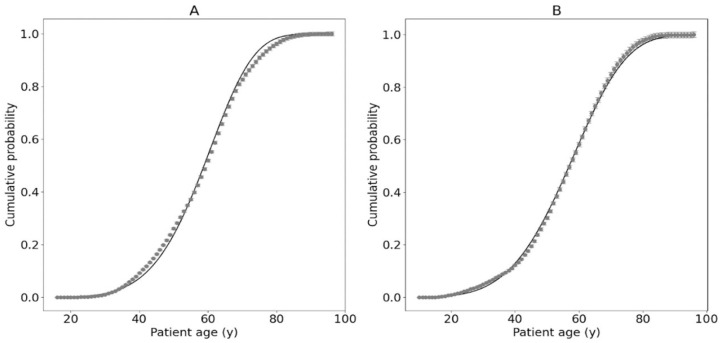
Cumulative probability of cancer diagnosis as a function of patient age at the time of diagnosis calculated with the Avrami–Dobrzyński model for the C50 group (**A**) and the C56 group (**B**). Grey points represent clinical data, while black lines represent model fit.

The clinical data for breast (C50) and ovarian (C56) patients are presented in Figure 2, together with the fitted Avrami–Dobrzyński model. Additionally, the parameters of the Avrami–Dobrzyński model for all the clinical data collected in the MSCI are shown in Table 1, together with the information about all groups of patients.

**Table 1:** Results of the application of the Avrami–Dobrzyński model to breast and ovary cancer clinical data received from the MSCI, divided into three datasets. All uncertainties represent one standard deviation.

No. of the Dataset	No. of Patients	Cancer Type	*α* Parameter (y^−1^)	*k* Parameter	Description
1	459	Breast, ovary, or both	(3.6 ± 1.6) 10^−11^	6.32 ± 0.12	Patients without *BRCA1/BRCA2* (no-*BRCA* group); see Figure 1
	52		(5.0 ± 6.0) 10^−10^	5.69 ± 0.33	Patients with *BRCA1/BRCA2* (*BRCA* group); see Figure 1
2	20,802	Breast	(4.16 ± 0.21) 10^−11^	5.775 ± 0.013	Patients diagnosed with breast cancer (C50 group); see Figure 3
3	9106	Ovary	(6.58 ± 0.43) 10^−9^	4.570 ± 0.016	Patients diagnosed with ovarian cancer (C56 group); see Figure 3

### 2.3. Protection Curve

An important finding can be observed in Figure 1: At the beginning of the plot (for women under 30 years old), there is a statistically significant difference between data points and the theoretical curve. This difference is regularly observed for various sets of data, except for the breast cancer data for men only.

**Figure 3:**
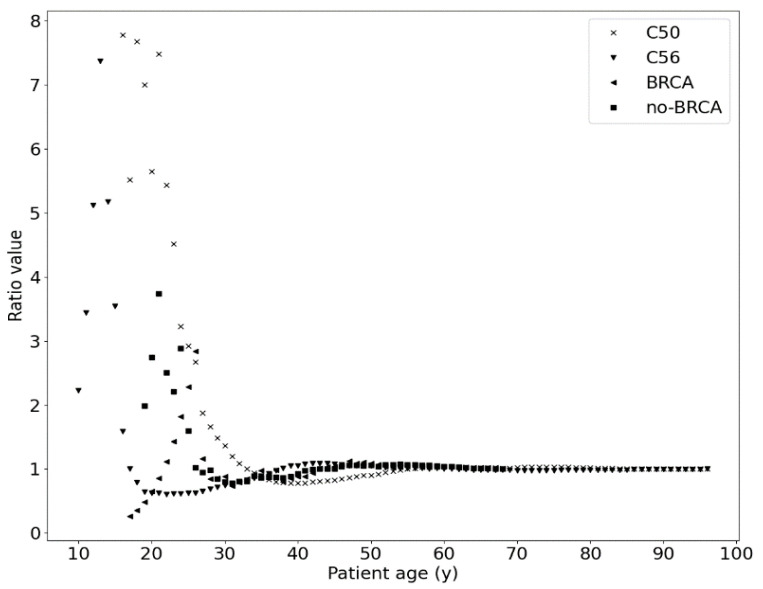
Ratio values between theoretical and experimental data as a function of patient age calculated for each clinical dataset (see Table 1). These functions represent potentially better immune protection for young women.

Figure 3 illustrates the ratio values between the theoretical and experimental data of Figure 1 and Figure 2 as a function of the age of the patient at the time of diagnosis. This is equivalent to the protection curve, which is responsible for the potentially better immune protection of young women. However, this finding is just a hypothesis.

**Figure 4:**
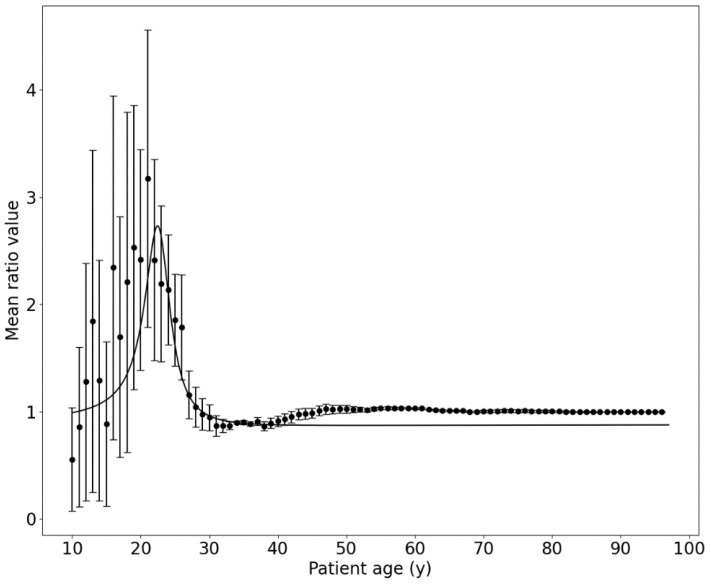
The average protection curve, *f*(*t*), represents the hypothetically better immune barrier for young women. Data points represent averaged values from Figure 3, while the black line is the best fit using the empirical function from Equation (3).

The average protection curve, *f*(*t*), was estimated as a mean value calculated for all curves in Figure 3, with the best fit (*R*^2^ = 0.76) using the following function (Figure 4):

(3) $$f\left(t\right)=\frac{p_{1}t^{2}+p_{2}t+p_{3}}{t^{2}+q_{1}t+q_{2}}$$

where *p*_1_ = (0.883 ± 0.050) y^−2^, *p*_2_ = (−40.6 ± 2.5) y^−1^, *p*_3_ = 482 ± 41, *q*_1_ = (−45.3 ± 3.5) y^−1^, and *q*_2_ = 518 ± 42. Other functions, like log-normal, Gaussian, or Weibull distributions, which were also tested as a best fit to the data points from Figure 4, had lower statistical likelihood than the one from Equation (3). One has to note that the shape of the function given by Equation (3) and presented in Figure 4 is a typical function of the adaptation of the organism to the external stressor [13].

The proposed protection curve can be treated as a correction function, which can be applied directly to Equation (2) and thus modify the Avrami–Dobrzyński model:

(4) $$P\left(t\right)=f\left(t\right)\cdot \left(1-e^{-\alpha \;t^{k}}\right)$$

This modified version of the model seems to be more appropriate to wider cancer data; however, it introduces a few more parameters.

### 2.4. Fractality

All the results presented show that the parameter *k* is always higher than 4. According to the Avrami nucleation and growth theory [15,16,17], the case in which *k* = 4 is equivalent to the three-dimensional development of the crystal in the environment. This means that for *k* > 4, this development has fractal dynamics. This was also confirmed for gastric cancer, where *k* = 4.42 ± 0.24 [14].

The weighted average value of the dimension parameter was equal to *k* = 5.30 ± 0.16 (based on all data from Table 1). This means a statistically significant fractality of cancer transformation up to the values obtained using the Avrami–Dobrzyński model. The open question is whether this finding is connected with the fractal geometry of DNA [25,26,27,28] or rather a multidimensional structure of complex protein metabolic network within the DNA of cancerous cells [29,30,31]. This will be discussed in the Section 3 below.

## 3. Discussion

‘Cancer is a robust state of living matter, which can be rephrased in terms of nonlinear systems as a stable attractor of a complex dynamical system that is represented by a living cell’—this very accurate statement written by Davies et al. [19] gives us an essential merit of the physics of cancer transformation. This process is, in fact, a phase transition between two states of living matter, which can be analogous to the phase transition in condensed matter physics or complex networks [32].

The Avrami–Dobrzyński model is an example of classical physical phase-transition theory implementation to the biological environment. This assumes that each mutated oncogene within the cell DNA is analogous to the crystallisation cluster (grain). This approach explains the rapid process of the cancer (neoplastic) transformation of mutated cells when the number of oncogenic mutations exceeds some effective threshold value. The dynamics of that process is, therefore, described using the sigmoidal function given by Equation (2), and the phase transition is of second order (the so-called continuous phase transition).

The original Avrami theory [15,16,17] is based on several fundamental assumptions, namely (a) the phase transformation starts with germ nuclei smaller than the critical size in the parent phase; (b) the initial number of germ nuclei decreases, and they are engulfed by growing grains of the new phase; (c) the growth rate of all grains is constant; (d) growth ceases at hard impingement but continues elsewhere until the entire system transforms; and (e) grains that could have formed if their nuclei had not coincided with an already-occupied area or had not been consumed by growing grains are termed ‘phantom grains.’ Making exceptions and adjusting some of these rules, the Avrami sigmoidal function can be simply applied to many other sciences, like genetics, cancer studies, biochemical applications, ecology, epidemiology, chemistry, social studies, and many other fields [18]. In the case of cancer physics, we assumed some significant changes to adapt the Avrami framework to the biological case [14]. This approach and the application of the target multi-hit theory present a new notion called the Avrami–Dobrzyński model of carcinogenesis.

The sigmoidal shape of the Avrami–Dobrzyński function is determined with the dimensionality factor, *k*. Each *k* value significantly higher than 4 represents the fractal dynamics of the phase transition (here, cancer transformation). This can be explained in two alternative ways:

- The geometric structure of DNA represents a fractal character; indeed, the DNA globule has many limited fractal elements, such as self-similarity and limited scale-free or power-law distribution of some DNA elements [25,26,27,28]. However, the process in which this fractality is functioning during cancer transformation is still unknown, especially from a dynamic point of view.
- DNA creates a complex multidimensional protein and metabolic network [29,30,31]; the proposed dimensionality problem should not be thought of as a spatial dimension related to the geometry of the DNA globule but rather the effective dimension of the network of protein interactions that are encoded by DNA. If we draw all proteins in the cell into a large network and connect all the nodes of proteins participating in the same processes (they have high affinity, regulate each other, etc.), we will obtain a network with a complicated topology. Such a complex network of connections can be associated with a dimension characterising the structure of this network (e.g., how many proteins are connected by one edge, how many by two edges, three, etc., and how it grows with the number of edges), not the space in which we draw it. In that way, the number of neighbours acts as a mathematical concept of dimension, and this feature does not have to be limited to 3D but can reach any number.

At this time, it is hard to determine which solution is accurate. However, this will be a matter of future research.

Another issue found within the presented study is a difference between the model and clinical data for patients under 30 years of age. The simple explanation is, of course, the strong limitation of the Avrami–Dobrzyński model, but this can be solved with a correction factor added to the original function; see Equation (4). This correction function has a typical shape of an adaptive response of the organism to some external stressor; see Figure 4. This means that—under the assumption of model correctness—the organism has an additional immunological protection mechanism or mechanisms that protect young women against cancer. It is worth noting that this phenomenon is not presented for men’s breast cancer cases, which were analysed separately. Therefore, this ‘protection mechanism’ can be connected with the fertility period in women, but it is a hypothesis only.

The immune system is strongly involved in the prevention of cancer development as the body’s primary defence pathway of the body. The cancer immune response mechanism involves immunoediting, which is based on the three-step concept of the inhibition of cancer cell development and growth: recognition, equilibrium, and elimination. Although the biomedical explanation for this phenomenon is still unknown, it has been observed that T-cell activity (the expression of cytokines and other molecules) plays a crucial role in these phases [33]. Most of the research focusing on the immune response with age points to immune senescence (which occurs in elderly patients) rather than specific mechanisms for young adults. Generally, it is stated that younger people have a robust (adaptive) immune system [34]. The protection that increases from childbirth to this stage stabilises, reaching its maximum, and then weakens with age [35]. This could explain the strongest efficiency of immune response at this point. In recent years, the number of observations on the effects of vaccination in this group versus older people only confirmed significant differences in the response of the immune system of these patients [36].

Current data suggest that genetic susceptibility explains up to 10% of all breast cancer cases [37]. Other risk factors are, for example, sex, obesity, or breast density [38]. Taking into account the 2–5-year period (30 divisions, double time, ~180 days depending on age) of tumour development, it is also possible that the physiological immaturity of (for example) the mammary gland can explain the lower probability of the values of breast cancer for adolescents and young women. However, it is worth mentioning that this group is more likely to have larger breast tumours with unfavourable characteristics and early distant metastatic or adverse outcomes. Additionally, the higher percentage of cases is due to genetic predispositions [39]. There are several more factors to consider above the immune response to fully understand an observation of the protection mechanism of young adults.

As can be seen in Table 1, the results show a ten-fold difference in values for the *α* parameter between the carriers of the *BRCA1/2* mutation and patients without this genetic burden. This observation is in agreement with the risk of developing breast/ovarian cancer, which is one order of magnitude different between the two considered groups. Therefore, this can mean that this parameter describes not only mutation distribution as in the case of the *a* parameter (from Equation (1)), but in the context of age-generated mutations, it involves the weights of specific gene variants in certain types of cancer. Having a similar dataset for other genetic predispositions tested would help us to confirm this supposition.

In conclusion, the application of the recently proposed Avrami–Dobrzyński model [14] works well for clinical cancer data. This model, validated for gastric cancer [14], shows that breast and ovarian cancer cases can be successfully described (and therefore predicted) for large populations of people for whom the exact number of oncogenic mutations is not precisely known. Indeed, because of that, one can create the risk function for patients (with or without *BRCA1/BRCA2* mutations) related to their age only and assess the risk of breast or ovarian cancer for individuals. This can help them and their physicians plan an appropriate process for cancer prevention, diagnosis, and, if necessary, therapy.

## 4. Materials and Methods

### 4.1. Clinical Data Collection

All clinical data on breast and ovary cancer were collected at the Maria Skłodowska-Curie National Research Institute of Oncology (MSCI) in Warsaw, Poland. All data were divided into 3 groups, and the total number of patients was 30,419. The first collection contains 511 cases of cancer patients with breast (429 cases), ovarian (55 cases), or both types of cancer (27 cases), from a previously published study [40]. The age of patients at the time of diagnosis was between 17 and 68 years old. The family history of cancer cases was registered for 457 patients (89.4%). In 52 cases *BRCA1* (28 patients) or *BRCA2* (23 patients) mutation was detected (*BRCA* group), while 459 cases were *BRCA1/BRCA2*-free (no-*BRCA* group) [40]. Mutations were tested with the NGS technique. Please note that one patient carried both mutations (*BRCA1* and *BRCA2*), so the total number of 52 cases resulted from 28 + 23 + 1.

For the second and third groups, the MedStream Designer software (MSD, ver. 4.2.0.1) was used for data extraction. Diagnosis data (ICD code) and patient age at the time of diagnosis were collected. Information about mutations was not available. The C50 group consisted of patients diagnosed with breast cancer during the time interval 11 June 2007–7 October 2022. The database contained 20,965 patients (20,802 female and 163 male); however, after the exclusion of males, the group of 20,802 was finally examined. The median age of diagnosis was 60 years (range 16–96). The cases were mainly unspecified breast malignant neoplasm (C50.9, 66%) or the malignant neoplasm of the breast (C50, 18%). The C56 group included 9106 patients with C56 (malignant neoplasm of the ovary) ICD diagnosis code. The data collection time interval was 2 January 1997–13 October 2022, and the median age of diagnosis was 58 (range 10–96).

### 4.2. Statistical Analysis

The data from cancer patients were aggregated as the age cumulative incidence distribution (probability). Next, the Avrami–Dobrzyński model was applied by fitting the function given by Equation (2) to the aggregated data. For fitting, the Levenberg–Marquardt algorithm, which was implemented in the Python library, was used [41,42,43]. Owing to this best-fit approach, it was possible to identify all model parameters (*α* and *k*) with their proper uncertainties. All uncertainties represent one standard deviation.

As mentioned earlier, dataset no. 1 is composed of two subgroups (with or without *BRCA1*/*BRCA2* mutations). This creates a time shift between both relationships (Figure 1). This time difference between both subgroups of data was calculated by fitting the modified *P*(*t* − *t*_0_) function (with additional time shift *t*_0_ parameter). 

The differences between the experimental data and the theoretical predictions of the Avrami–Dobrzyński model were estimated using ratio calculations. This was a basis for the protection curve assessment.

## 5. Conclusions

The presented paper describes the practical application of the novel biophysical model of cancer induction in patient populations. The proposed approach is based on the purely physical concept of phase transition, which is an analogy to cancer transformation. On this basis, clinical data from breast and ovarian cancer patients (from the Maria Skłodowska-Curie National Research Institute of Oncology, Warsaw, Poland) were used in the framework of the Avrami–Dobrzyński model of carcinogenesis. The clinical data were divided into three datasets due to the differences between them, and the model parameters were calculated. These results can be useful for the prediction of cancer risk for patients and their physicians. For example, patients with hereditary *BRCA1/BRCA2* mutations can be diagnosed with cancer approximately 2 years earlier than patients without such mutations (see Figure 2). This suggests the hypothesis that a new oncogenic mutation can be obtained every two years of human life in breast or ovarian somatic cells (assuming a linear relationship between such mutations and age). Furthermore, it should be noted that the results suggest that carcinogenic processes follow the fractal dynamics with the average Avrami dimension factor equal to *k* = 5.30 ± 0.16. However, the source of this fractality is still unknown. Finally, we found that patients younger than 30 years of age exhibit additional immune protection against cancer, which is represented as the difference between the model prediction and the clinical data.

## Data Availability

Data is contained within the article.

## Abbreviations

ATM	Ataxia–telangiectasia mutated
BOC	Breast and ovarian cancer
BRCA	Breast Cancer gene
CDH1	Cadherin1
CHEK2	Checkpoint kinase 2
DNA	Deoxyribonucleic acid
ICD	International Classification of Diseases
JMAK	Johnson–Mehl–Avrami–Kolmogorov
MSCI	Maria Skłodowska-Curie National Research Institute of Oncology
MSD	MedStream Designer
NBN	Nijmegen breakage syndrome (nibrin)
PALB2	Partner and localizer of BRCA2
PTEN	Phosphatase and tensin homolog
TP53	Tumor protein p53
XRCC2	X-ray repair cross-complementating 2
